# Application of Eugenol in Poultry to Control *Salmonella* Colonization and Spread

**DOI:** 10.3390/vetsci10020151

**Published:** 2023-02-13

**Authors:** Mohammed Aljuwayd, Israa Abdullah Malli, Young Min Kwon

**Affiliations:** 1 Cell and Molecular Biology (CEMB), University of Arkansas, Fayetteville, AR 72701, USA; 2 College of Medical Applied Sciences, The Northern Border University, Arar 91431, Saudi Arabia; 3 College of Medicine, King Saud bin Abdulaziz University for Health Sciences, Jeddah 21423, Saudi Arabia; 4 King Abdullah International Medical Research Center, Jeddah 22384, Saudi Arabia; 5 Department of Poultry Science & The Center of Excellence for Poultry Science, Fayetteville, AR 72701, USA

**Keywords:** poultry, *Salmonella*, salmonellosis, chicken manure, multi-drug resistant strains, *Salmonella* Enteritidis, *Salmonella* Typhimurium, eugenol

## Abstract

**Simple Summary:**

The poultry sector is an essential component of agriculture that has grown unusually over the past few decades. *Salmonella* is thought to live naturally in chickens and causes salmonellosis in humans. Many plant extracts, mainly essential oils, have had their active ingredients studied. *Salmonella* in chicken is resistant to the antimicrobial effects of the aromatic oil eugenol, which is present mainly in cinnamon and clove. Therefore, eugenol supplementation also improves gut health, thereby increasing overall well-being. Here, we reviewed the rising rates of salmonellosis worldwide and the factors contributing to its prevalence. Then, we suggested using eugenol as a natural feed supplement for reducing *Salmonella* in chicken.

**Abstract:**

The poultry sector is an essential component of agriculture that has experienced unprecedented growth during the last few decades. It is especially true for the United States, where the average intake of chicken meat increased from 10 pounds (4.5 kg) per person in 1940 to 65.2 pounds (29.6 kg) per person in 2018, while the country produced 113 billion eggs in 2019 alone. Besides providing nutrition and contributing significantly to the economy, chicken is also a natural reservoir of *Salmonella*, which is responsible for salmonellosis in humans, one of the significant foodborne illnesses around the globe. The increasing use of chicken manure and antibiotics increases the spread of *Salmonella* and selects for multi-drug resistant strains. Various plant extracts, primarily essential oils, have been investigated for their antimicrobial activities. The multiple ways through which these plant-derived compounds exert their antimicrobial effects make the development of resistance against them unlikely. Eugenol, an aromatic oil primarily found in clove and cinnamon, has shown antimicrobial activities against various pathogenic bacteria. A few reports have also highlighted the anti-*Salmonella* effects of eugenol in chicken, especially in reducing the colonization by *Salmonella* Enteritidis and *Salmonella* Typhimurium, the primary *Salmonella* species responsible for human salmonellosis. Besides limiting *Salmonella* infection in chicken, the supplementation of eugenol also significantly improves intestinal health, improving overall well-being. In this review, we highlight the rising incidences of salmonellosis worldwide and the factors increasing its prevalence. We then propose the usage of eugenol as a natural feed supplement for containing *Salmonella* in chicken.

## 1. Introduction

Foodborne illnesses are one of the major causes of contributors to disease burden worldwide [1]. In the United States (US) alone, approximately 48 million people each year contract a foodborne disease, which costs around $90 billion to the US economy in healthcare expenses and reduces the economic activities of the affected individuals. Among many other foodborne pathogens, *Salmonella* from poultry is estimated to cause $2.8 billion in financial losses to the US [2,3]. *Salmonella* bacteria have emerged as the leading etiological agent of foodborne illnesses worldwide, posing a serious threat to public health. A significant number of foodborne infections worldwide are confirmed to be caused by *Salmonella*, which produces various disease symptoms collectively referred to as salmonellosis [4]. Because of the severe implications of *Salmonella* to public health, control of this pathogen in poultry has become a top priority of the US Department of Agriculture [USDA] [5]. *Salmonella* serovars have been shown to infect various domesticated animals, including cattle, poultry, pigs, and sheep. Infected animals may exhibit symptoms ranging from mild gastroenteritis to death in extreme cases of *Salmonella* infection [6]. Most cases of salmonellosis are attributed to eggs and chicken meat consumption. The human condition is commonly caused by a few serotypes, such as *Salmonella* Enteritidis (*S*. Enteritidis) and *Salmonella* Typhimurium (*S*. Typhimurium), with poultry serving as a primary reservoir [7,8]. However, there has been a recent increase in the prevalence of multidrug-resistant (MDR) *Salmonella* enterica [9]. Pietsch M. et al. have reported third-generation cephalosporin resistance phenotypes resistant to three or more antimicrobials [10].

The incidence of *Salmonella* infection in poultry varies by country. In some industrialized countries, roughly 1% of the poultry flock is infected with *Salmonella*. On the other hand, resource-poor countries can have as much as a 10% *Salmonella* infection rate in chickens due to challenging epidemiological settings [11]. The spread of pathogens originating in the chicken host, especially *Salmonella*, can spread faster and more efficiently due to the reuse of litter-containing bedding material and chicken droppings. Chicken producers increasingly use litter in farming due to the increased cost and difficulty procuring bedding material [12,13].

Moreover, chicken droppings are also extensively used as fertilizer due to the abundance of nitrogenous materials in the chicken feces. A study by Chinivasagam et al. [14] found that the farms that reuse litter had as much as 83% *Salmonella* prevalence compared to 68% in farms that disposed of litter after a single use by the flocks. Even if the manure is not reused and disposed of, the persistence problem of *Salmonella* still exists, as evidenced by the high prevalence of *Salmonella* in farms that regularly dispose of the chicken litter containing manure, as shown by Chinivasagam et al. [14]. Hence, research into effective ways of eradicating *Salmonella* from poultry is urgently needed to reduce the burden of salmonellosis and the economic destruction caused by it to the poultry industry and public healthcare [15].

Various management and treatment strategies have been proposed to reduce *Salmonella* infection and spread in chickens. Among these approaches, the pre-slaughter method is a primary method for controlling *Salmonella* in chickens [16]. The main goal behind this strategy is to reduce pathogen colonization in poultry through various intervention strategies, which can ultimately lead to a decrease in poultry meat and egg contamination. Various preslaughter approaches have been used over the years, such as vaccination, competitive exclusion of *Salmonella* in chickens by modulating gut bacteria, and the use of probiotics and prebiotics [17]. These techniques have shown varying levels of success in controlling *Salmonella* infection and colonization in chickens [18]. Various plants having therapeutic value have been used in animal and food production for centuries. These plants, especially their bioactive compounds and essential oils (EOs), are beneficial in containing *Salmonella* infection in chickens [19]. Secondary compounds produced by plants confer the main therapeutic action against pathogens. Such compounds are produced as byproducts of the interaction between plants and their environment [20]. 

The main advantage of plant-based compounds over antibiotics is multi-faceted antimicrobial action, as opposed to the limited antimicrobial mechanisms of antibiotics. The likelihood of developing bacterial resistance against plant-based antimicrobials is minimal [21]. EOs are one of the most potent plant-based resources, and among the essential oils, carvacrol, thymol, and eugenol have shown promising potential against *Salmonella* infections in chickens. Eugenol, a primary component of the EOs extracted from various plants but present in abundant amounts in clove, has been shown to kill many enteric pathogens, including *Salmonella*, selectively [22,23]. Moreover, one of the main advantages of plant-derived compounds is their non-destructive effects on the commensal microbiota in chickens. Therefore, plant-based extracts, especially EOs, can selectively kill various enteric pathogens without causing much harm to the gut commensal bacteria [22,24]. In the current review, we provide information on the destructive effects of *Salmonella* and the use of antimicrobials that increase this pathogen’s persistence. We then highlight the potential application of eugenol as an alternative to antibiotics for treating *Salmonella* in chicken. 

## 2. Effect of Salmonellosis on Humans

*Salmonella* is a flagellated, facultative anaerobic, gram-negative bacillus. The genus belongs to *Enterobacteriaceae*, a large group of medically important enteric pathogens. *Salmonella* species are found in the microbiota that colonizes some humans and animals [25]. Salmonellosis is a zoonotic illness caused by *Salmonella* species, affecting humans and causing severe complications. It is a very common foodborne illness around the world. In the United States, the Center for Disease Control (CDC) estimates that salmonellosis affects about 1.2 million people and causes 450 deaths yearly [26].

Moreover, salmonellosis affects humans and farm animals, since *Salmonella* is endogenous in some animals, causing substantial livestock losses. Besides livestock, the most common animal reservoirs are birds, such as chickens, ducks, and turkeys [27]. *Salmonella* contamination of cattle and poultry may come through feed. The trends in animal feed and feed additives are tracked in the US by the FDA Center for Veterinary Medicine. *Salmonella* contamination of feed and making animals ill can also harm humans through direct contact with contaminated feed or through infected animals shedding the bacteria into human food and water supplies [28]. In humans, salmonellosis manifestations depend on the causative agents and the immune regulation inside the host. It can manifest as an asymptomatic carrier, gastroenteritis, enteric fevers, dehydration, arthritis, and septicemia. Nontyphoidal salmonellosis, caused by *S.* Enteritidis and *S.* Typhimurium, is associated with food poisoning cases [29]. 

It has been estimated that as high as 41% of retail chicken meat can contain *Salmonella*; this percentage is considered second after *Campylobacter*, the most prevalent bacterial pathogen in chicken, with an estimated prevalence of 70.6% [30]. Besides meat, chicken eggs are also a major source of the spread of salmonellosis [31]. It has been estimated that one in 20,000 eggs carry *Salmonella*. This might not sound like much, but the per capita consumption of chicken eggs is much higher than that of chicken meat, which makes it a significant source of *Salmonella* transmission to the public [32]. One of the major contributors of *Salmonella* in chicken products is thought to be fecal contamination. Because of this, the US Food Safety and Inspection Service (FSIS) has enacted mandatory inspection procedures for fecal contamination of poultry products for zero tolerance of fecal material in chicken meat and eggs. These procedures include the routine inspection of *Salmonella*, *Campylobacter*, and *Escherichia* species in chicken products [33].

## 3. Use of Antibiotics to Contain Salmonella in Chicken

The poultry sector has grown exponentially since the industrialization of poultry farming in the last century and is expected to grow further. The progress made in the chicken industry is attributed to multiple factors. The therapeutic usage of antibiotics, chicken vaccinations against communicable illnesses, organic nutrition, a healthy breeding environment, and gene selection are some of the contributing factors [34]. The success of this sector can in part be attributed to the use of antibiotics, which have been used at therapeutic and sub-therapeutic levels to protect the chicken from infectious diseases and enhance their economic output. Antibiotics help poultry birds mature and expand their body size by improving feed conversion capacity and minimizing disease occurrences [34,35].

Furthermore, antibiotics improve general animal well-being, and combining all these factors minimizes the cost of production associated with producing animals. As a result of the financial benefits of adding antibiotics to feed, food output improves, which is shared across value-added chains. Most cost savings associated with using antibiotics in animal agriculture are due to better feed conversion, improved litter and manure quality, and reduced mortality [36]. Despite these benefits, the ongoing use of antibiotics in chicken production generates public health and safety concerns about antimicrobial resistance and antibiotic residue accumulation in the environment and food supply [36,37]. The overuse of antibiotics in animal farming leads to the selection of antibiotic-resistant (ABR) genes in the bacteria residing in animals. These ABR genes can easily spread to bacterial populations in the environment, plants, and animals, including humans [38].

The spread of ABR is associated with increased medical care costs and mortality rates in humans due to increased antibiotic resistance. The European Union estimated that antibiotic-resistant infections cost around $1.5 billion, while around 25,000 people die yearly from these antibiotic-resistant bacterial infections [36,39]. However, suppose the increase in antibiotic resistance continues. In that case, it is feared that we will enter a post-antibiotic era that could cause the deaths of 10 million people by 2050 within a few decades. The high usage of antibiotics is also selected for the emergence of multidrug-resistant (MDR) *Salmonella* strains. The poultry sector is also the most significant contributor to the emergence of MDR *Salmonella* strains. It has been shown by many studies that most MDR *Salmonella* strains isolated from poultry belong to *S.* Enteritidis and *S.* Typhimurium, the causative agents of salmonellosis in humans [40]. There was a reported increase in the prevalence of multidrug-resistant (MDR) *Salmonella* enterica species, such as the third-generation cephalosporin resistance phenotypes and MDR-ACSSuT *Salmonella* enterica, which was reported to be resistant to ampicillin, chloramphenicol, streptomycin, sulphonamides, and tetracyclines. The resistance mechanisms were linked to int1-associated elements and extended-spectrum β-lactamase (ESBL) genes [9,10,40].

Moreover, the extensive use of antibiotics also destroys the chickens’ normal gut microbiota, leading to an imbalance in the microbial diversity of the chicken gastrointestinal (GI) tract. This disturbance of microbiota, called gut dysbiosis, causes a disturbance in the immune system and makes chickens vulnerable to various infectious and non-infectious diseases [36,37]. Thus, problems with the use of antibiotics in poultry farming are compelling scientists to discover reliable, cost-effective, and efficient means that could be used as an alternative to antibiotics in animal production. There are several alternatives proposed over the years. Various plants and their derivates are extensively studied due to their antioxidant, antiparasitic, anti-inflammatory, antiviral, and antibacterial activities [38,39].

## 4. Use of Chicken Manure in Poultry Farming and Spread of *Salmonella*

Animal manure is an excellent source of energy and nutrients; therefore, it is increasingly being used in agriculture and animal production, including poultry farming. However, besides containing these desirable features, animal manure is also rich in harmful pathogens and ABR genes. Chicken manure is untreated animal feces commonly used for agricultural and gardening [40,41]. It makes an excellent fertilizer that improves soil structure, moisture-holding capacity, and water infiltration. It is considered a cost-effective source of nitrogen, potassium, and phosphorus. Even though chicken excreta has several advantages, raw chicken manure fertilizer can transmit serious human infections [42]. Chickens are mostly reared on litter that contains bedding material and chicken feces or manure. Chicken manure contains various pathogens (many zoonotic), including *Salmonella*, which can spread easily between chickens in a flock as they all share common living conditions within a poultry farm. This increases the incidence of infectious diseases in chickens and humans who consume or encounter them [12,43]. Yang et al., 2016, investigated the effects of chicken manure on microbiome communities. Using genomic DNA analysis, the authors detected an increase in the soil’s pathogenic antibiotic-resistant bacteria population. Therefore, the authors demonstrated a potential threat to human health because of the improper usage of chicken excreta (Yang et al., 2016). Since *Salmonella* colonizes the gut of chickens, many outbreaks have been documented, related to improper chicken manure usage and handling of plant products [44]. Thus, increasing usage in various sectors can accelerate the spread of pathogens and ABR genes [45,46].

The factors that contribute to the spread of *Salmonella* in chicken manure include temperature, moisture, and litter usage [47]. Increased moisture and litter re-usage increases *Salmonella* prevalence in chicken manure, while temperatures unsupportive of the growth of *Salmonella* are associated with decreased *Salmonella* counts [14,47]. Even in the chickens infected with *Salmonella* strains, heterogeneity still exists in their ability to release *Salmonella* into the environment or transmit bacteria to other organisms [48]. The infected chickens that harbor and spread the *Salmonella* bacteria at higher levels than other chickens in the flock are called super-shedders for *Salmonella*. The trait can be found in genetically homogenous populations of host organisms, as shown by studies in inbred mice [49] and chickens [50]. Thus, factors other than host biology can also determine whether a host will be a super-shedder [48]. The transmission rate of these super-shedders makes them a focus for epidemiological studies and disease management. Empirical studies and modeling for numerous diseases have shown that 20% of individuals infected with pathogens contribute to 80% of the spread of any infection [51]. Therefore, strategies are required to decrease the transmission rate of *Salmonella* by chicken, especially super-shedders. There is insufficient information regarding what makes super chicken shedders for these bacteria. Hence, further research is required to determine the underlying reasons [48]. One factor contributing to the colonization and shedding of *Salmonella* in chickens includes gut microbial composition. Therefore, modulating the gut bacteria of chickens can help in decreasing the colonization of *Salmonella* in chickens and the eventual spread of this bacterium through feces [47].

## 5. Gut Microbiota in Chickens

Chickens acquire their microbiota shortly after birth, which is formed initially from the microorganisms inherited from mother hens and through the contamination of eggs by the environment [52]. This microbial community changes throughout the lifetime of chickens and is impacted by the nutritional intake, the environment surrounding chickens, and the sex and breeds of chickens [53]. The gut of chickens contains many microbial species dominated by bacteria [54]. It has been estimated that an individual chicken contains around 100 billion bacterial cells in the GI tract, far more than in any other organ of a chicken’s body. Most chicken GI tract bacteria are anaerobes, which can be obligatory and facultative. In a healthy chicken gut microbiota without dysbiosis, beneficial gram-positive bacteria predominate (as much as 85% of the total microbiome can contain gram-positive bacteria). Even in healthy chicken, *Salmonella*, other bacteria such as *Escherichia coli* (E. coli) and *Campylobacters*, can be present, but in much lower amounts than in the diseased chicken [55]. The gut microbiota can affect chicken physiology by contributing to plant polysaccharide digestion, toxin metabolism, and immunity against pathogens [56,57]. The gut microbiome also contributes to the chicken body mass [58]. This is mainly due to the involvement of microbiota in the generation of nutrients vital for chicken growth, such as amino acids, fatty acids, for example, short-chain fatty acids (SCFAs), vitamins, and ammonia [59].

The colonization of microbiota in the chicken gut also modulates the shape of the GI tract. It helps stimulate chickens’ innate and adaptive immune responses that help the host fight a foreign pathogen. Aside from GI tract shape modulation and immune stimulation, the gut microbiota, due to their advantage of inhabiting the host body earlier and adapting to it, resist the incoming bacteria from permanently colonizing the host. This mechanism is called competitive exclusion, one of the main ways the host body eliminates foreign pathogens [60]. An imbalance in the gut microbiota can cause diseases, low productivity and mass gain, and high mortality in chickens [61]. Several studies exist on the gut microbiota diversity and susceptibility to *Salmonella* infection in poultry [54,62,63]. Recently, a study by Litvak et al. [64] has shown that one of the commensal bacteria in the chicken gut (i.e., *Enterobacteriaceae*) protects neonate chickens from *Salmonella* infection by creating an oxygenated environment. The high oxygenation levels contain the colonization of *Salmonella* as they are toxic to the bacterium [64].

In most cases, infection with *Salmonella* does not follow any visible symptoms in chickens, and chickens remain asymptomatic carriers of *Salmonella* throughout their life. However, it has been experimentally shown that *Salmonella* infection causes suppression of the immune system, especially by increasing Treg cells in chicken, which increases their susceptibility to acquiring a microbial infection [65]. Therefore, it is important in the poultry industry to maintain a healthy microbiome in the chicken gut.

## 6. EOs and Their Usage in Chicken Farming

Using plant bioactive compounds in animal feeds has recently gained much attraction. These bioactive plant compounds are also called phytochemicals, phytogenics, or phytobiotics. When ingested into the body, these nutritional compounds are supposed to provide health benefits. A variety of such phytochemicals, when incorporated into animal feed, have been shown to increase body weight and decrease incidences of infectious diseases due to their antimicrobial activities and gut modulation in the animals ingesting such feed [66]. EOs are one of the most potent extracts of plants that can contain a variety of important phytochemicals from the plant. The most common methods for the extraction of EOs from plants include solvent extraction, steam distillation, and hydro distillation [67]. These plant extracts can be either terpenes or phenylpropenes based on the presence of these two major chemical compounds [68]. The two most accepted effects of EOs on the poultry gut include the stimulation of the secretion of digestive enzymes and the promotion of a favorable intestinal microbiota ecosystem. These effects increase feed utilization, reduce the risk of growth-depressing disorders, and decrease susceptibility to infections [69]. The antimicrobial effects of various EOs are generally well-studied. EOs are hydrophobic and work by partitioning the bacterial cell membrane’s lipophilic interior, causing the bacterial cells to burst and leak their cellular contents into the environment [22]. There are currently around 3000 EOs, with about 300 of them, including clove oil, being economically important [67].

### 6.1. Eugenol Oil from Clove, the Active Substance

**Figure 1:**
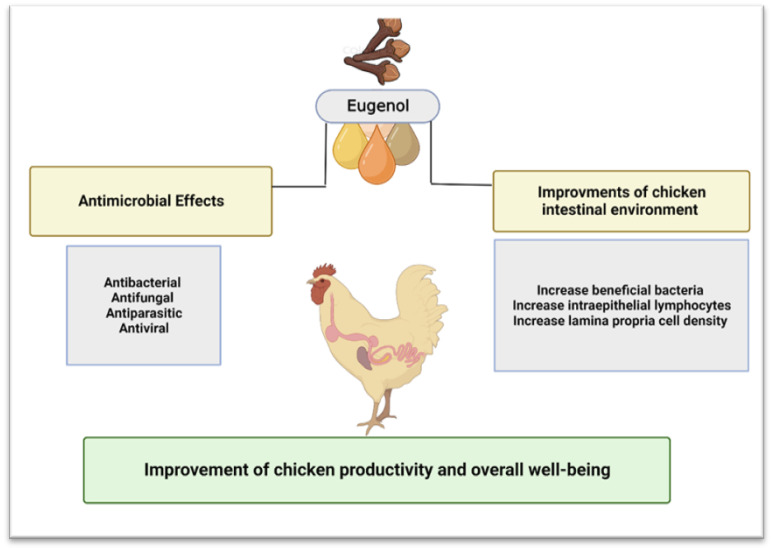
Beneficial effects of eugenol supplementation in chickens. Created with BioRender.com, accessed on 5 December 2022.

Eugenol, known chemically as 4-allyl-2-methoxy phenol, is an essential aromatic oil obtained from various plant sources, such as basil, bay, cinnamon, cloves, ginger, nutmeg, pepper, thyme, turmeric, and tulsi. However, only clove and cinnamon were shown to contain considerably high amounts of eugenol [70]. The oil is present mostly in the buds of cloves, which can contain eugenol in the range of 49 to 90%. On the other hand, eugenol is found mostly in the barks of cinnamon, which can contain 20 to 50% eugenol [71]. This EO has many beneficial effects on different body systems [72]. It belongs to a class of phenylpropenes essential in providing flavor and scent to spices and herbs containing this compound. Eugenol has been demonstrated to function primarily as a pollinator attractant for plants and an antibacterial, antiparasitic, antiviral, and antifungal compound [68]. Pramod et al. [59] also documented that eugenol acts as a significant antioxidant, preventing cellular inflammation, and showed its analgesic effects on local tissues [73]. Eugenol is structurally smaller than many other compounds that have actively been used and identified as antimicrobials; therefore, it may provide new mechanistic insights. According to Mahboub [72], eugenol is soluble in alkaline aqueous solutions and was approved in the human diet. It can be assessed as a safer candidate for studying its antimicrobial properties [72]. According to the Research Institute for Fragrance Materials (RIFM), daily intake of eugenol less than 300 mg/kg/day is safe for consumption and poses no toxicity to the consumer [74]. However, for chickens, the recommended eugenol amount is 100 mg/kg/day according to the Additives and Products or Substances used in Animal Feed (FEEDAP) report of the European Food Safety Authority [EFSA] [75]. The beneficial effects of eugenol supplementation in chicken farming (studied and those that can be studied) are shown in Figure 1 and discussed in the further subsections.

#### 6.1.1. Beneficial Effects of Eugenol on Chicken Gut

Supplementation of eugenol in chicken feed, alone or in addition to different plant extracts, has been previously shown to decrease pathogen counts, reduce disease severity by improving intestinal health, and increase the growth performance of chickens [76]. The beneficial effects of eugenol are related to a significant increase in the abundance of beneficial bacteria like *Lactobacilli*, immune cells, and intestinal epithelial cells, such as intraepithelial lymphocytes and lamina propria, respectively [77]. The *Lactobacilli* species are one of the most commonly known probiotic species employed in animal production. In the chickens, the strains belonging to *Lactobacilli* reduce *Salmonella* colonization, which ultimately reduces the latter’s fecal shedding [78]. Moreover, an increase in several lymphocytes is associated with improved pathogen clearance, while an increase in lamina propria cell density could help increase *Lactobacilli* numbers [77,79]. The beneficial effects of eugenol are also corroborated by research in mice. Eugenol administration through drinking water was reported to alter microbiota diversity in the colon and increase inner mucus thickness [80].

#### 6.1.2. Antifungal, Antiparasitic, and Antiviral Effects of Eugenol

Currently, there are no published reports on the antifungal, antiviral, and antiparasitic effects of eugenol in chickens. However, the positive effects of eugenol against the fungi, viruses, and parasites that cause human diseases are well established. Carrasco et al. [81] showed the antifungal activities of eugenol against three human opportunistic pathogen yeasts, namely *Candida albicans*, *C. neoformans*, and *Saccharomyces cerevisiae*, with MIC values ranging from 125 μg/mL to more than 256 μg/mL [81]. During the 2014 Ebola virus outbreak in West Africa, Lane et al. [82] investigated the antiviral activity of Eugenol against Ebola and other viruses. The Ebola virus is the causative agent of fatal hemorrhagic fever in humans. The authors reported that eugenol had shown antiviral activity against Influenza A and Herpes Simplex virus types 1 and 2. Thus, the authors proposed using this natural product against the Ebola virus [82]. Eugenol has also been tested against parasites. Raja et al. [83] screened the inhibitory effect of eugenol derivatives on *Leishmania Donovan*, the causative agent of visceral leishmaniasis, a life-threatening human parasitic infection. Eugenol derivatives showed strong parasitic activity against this disease, inhibited leishmanial replication, and cleared the infection completely from liver cells [83]. The therapeutic effects of eugenol in humans against these pathogenic organisms can be replicated in chickens to investigate the therapeutic usage of eugenol against chicken fungal, parasitic, and viral diseases.

#### 6.1.3. Antibacterial Effects of Eugenol

The antibacterial activities of eugenol have particularly captivated the attention of various scientists. The free hydroxyl groups in EOs confer antibacterial effects, and eugenol’s antibacterial properties are also due to its free hydroxyl group. This free hydroxyl group disrupts the bacterial cell membrane by altering the fatty acid composition, increasing the reactive oxygen species (ROS) concentration, or altering enzymatic activities. Moreover, the DNA damage effects of eugenol in bacterial cells have also been observed [84]. Recently, eugenol has been shown to decrease the motility and expression of molecules associated with pathogenicity and host cell attachment in chickens [85]. Eugenol has shown antibacterial activity against a variety of Gram-positive bacteria, such as *Bacillus cereus*, *Bacillus subtilis*, *Enterococcus faecalis*, *Staphylococcus epidermidis*, *Staphylococcus aureus*, *Streptococcus pyogenes*, *Streptococcus pneumonia*, *Listeria monocytogenes*, and Gram-negative bacterial species, such as *E. coli*, *Helicobacter pylori*, *Proteus Vulgaris*, *Salmonella choleraesuis*, *S. Typhi*, *S. Typhimurium*, *S.* Enteritidis, and *Yersinia enterocolitica* [70,85,86,87]. Eugenol also has been shown to possess biofilm-eradicating capacity. Biofilms are one of the most resilient bacterial structures that allow bacteria to resist the harshest conditions, including the availability of antibiotics in the environment. At a concentration of 0.5 × the minimum inhibitory concentration (MIC), 50 percent inhibition was observed in biofilms generated by methicillin-resistant *Staphylococcus aureus* (MRSA) and methicillin-susceptible *Staphylococcus aureus* (MSSA). However, combining carvacrol with eugenol lowered the minimum biofilm-eliminating concentration (MBEC) of already-formed biofilms to 99 percent, suggesting the synergistic effect of two EOs against bacterial biofilms [83]. Moreover, eugenol can also decrease the biofilm formed by *S*. Typhimurium by more than 50%, while the combinational use of cinnamaldehyde and eugenol increases antibiofilm activity against *S*. Typhimurium by 70% [88].

##### Anti-Salmonella Effects of Eugenol on Chickens

A handful of studies have reported the anti-*Salmonella* effects of eugenol. Based on a literature search, the scientific community’s interest in using eugenol for containing *Salmonella* in poultry is increasing. Incorporating eugenol in poultry feed has been shown to reduce *S*. Enteritidis colonization [89]. Moreover, Devi et al. [90] concluded that eugenol induced complete inhibition and reduced the viability of *S*. Typhi. The authors proposed that the main mechanism of action of eugenol against *Salmonella* was targeting the bacterial cell membrane [90]. Eugenol disrupts the bacterium’s cell membrane, which leads to the disruption of ATP synthesis, and the bacterial cells die due to the lack of energy [91]. In an in vitro chicken cecum model, eugenol treatment resulted in more than a five log^10^ CFU/mL decrease in *S*. Enteritidis bacterial cell counts [92].

The in vivo chicken models of eugenol supplementation have also shown promising results. It has been shown by Kollanoor-Johny et al. [86] that the addition of eugenol at 1% in chicken feed can significantly reduce (*p* < 0.05) the population of *S*. Enteritidis (>four log^10^ cfu/g) to 1.5 log^10^ cfu/g and two log^10^ cfu/g in the chicken cecum and cloaca, respectively. The supplementation of chicken feed with 0.75 and 1% eugenol significantly reduces (*p* < 0.05) *S*. Enteritidis counts in the chicken cecum (at least three log^10^ CFU/g or more *S*. Enteritidis reduction), which leads to reduced fecal shedding of the pathogen. The reduction in *S*. Enteritidis could be attributed to the eugenol-elicited reduced expression of genes involved in the invasion of chickens and the genes involved in metabolism, motility, the type III secretion system (T3SS), outer membrane proteins, and electron acceptor proteins [93,94]. Interestingly, supplementing feed with eugenol reduces chicken body weight gains (*p* < 0.05). This reduced weight gain could be due to reduced feed intake as the chickens with the eugenol-supplemented feed had significantly reduced (*p* < 0.05) feed intake in comparison with that of the control chicken groups [94]. One explanation for reduced feed intake due to eugenol or other EO supplementation is reduced palatability based on the strong smell and flavor. However, poultry shows tolerance for the moderate addition of EOs to feed [69]. Thus, other factors might have caused reduced feed intake, which might not have been considered by Kollanoor-Johny et al. [94].

**Figure 2:**
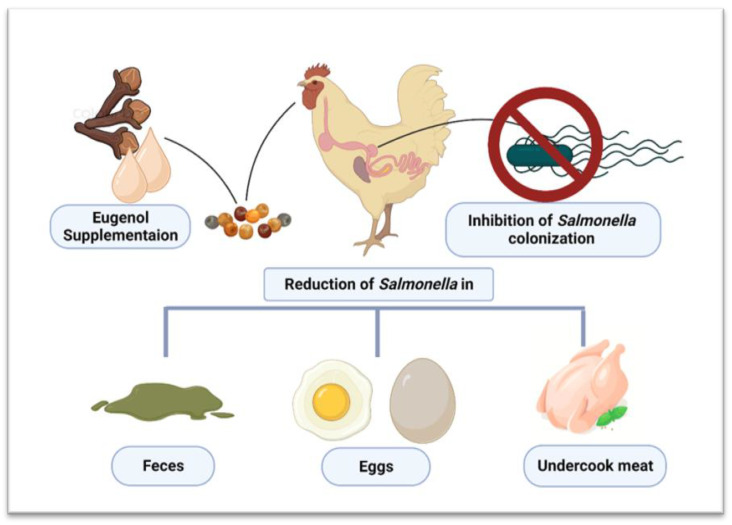
Effect of Eugenol supplementation in controlling *Salmonella* colonization in chickens and its spread. Created with BioRender.com, 5 December 2022.

Eugenol severely limits the motility of *S*. Typhimurium due to its destructive effects on fimbriae and decreases the expression of adhesion molecules and virulence factors. This not only decreases pathogenicity but also significantly reduces the *S*. Typhimurium counts in chickens, as shown by Zhao et al. [85], who reported a highly significant (*p* < 0.01) reduction of *S*. Typhimurium cells due to eugenol pretreatment. These bactericidal and bacteriostatic effects were due to the inhibition of the T3SS and mannose-sensitive type I fimbriae (TIF)-related adhesion virulence factors. The T3SS is a needle-like specialized apparatus capable of transporting effector proteins to host intestinal epithelial cells, critical for *Salmonella* pathogenicity. On the other hand, TIF is vital for the adherence of *S*. Typhimurium to host cells. Therefore, attenuating these genetic components lowers the pathogenicity and survivability of *S*. Typhimurium in chickens [67]. A summary of all studies performed on the anti-*Salmonella* effects in chickens is presented in Table 1. In contrast, an overview of the reduction of *Salmonella* colonization inside chicken guts is shown in Figure 2.

## 7. Conclusions

Salmonellosis is a zoonotic disease that is caused by the *Salmonella* bacterium. Poultry serves as one of the main reservoirs of *Salmonella* and is a significant source of its transmission to humans and the environment. In humans, *Salmonella* infections are responsible for salmonellosis, a disease that is among the most prominent foodborne zoonotic diseases. With the increasing use of chicken manure and antibiotics in the poultry industry, incidences of salmonellosis are expected to rise even further. Eugenol has the potential to serve as a safe alternative to antibiotics in poultry production for containing salmonellosis. Eugenol has been shown to improve the secretion of digestive enzymes and the overall diversity of the chicken gut microbiota.

Moreover, in vitro and in vivo models of *Salmonella* (especially of *S*. Enteritidis and *S*. Typhimurium, the causative agents of salmonellosis in humans) infection in chickens have shown the beneficial effects of eugenol. However, there is still a lack of mechanistic studies describing how eugenol supplementation reduces *Salmonella* in chickens. Moreover, only the effect of eugenol on *Salmonella* strains that cause salmonellosis in humans has been studied, highlighting the need to investigate eugenol’s effect on poultry disease-specific strains, such as *S*. Pullorum and *S*. Gallinarum as well. Both of these strains are one of the major causes of economic destruction to the poultry industry [83]. Therefore, studies exploring the molecular mechanisms of eugenol targeting *Salmonella* inside the chicken body and the therapeutic efficacy of eugenol against poultry-specific *Salmonella* strains are needed. Moreover, attention must also be paid to investigating the metabolic changes accompanying eugenol intake inside the bodies of chickens.

## Figures and Tables

**Table 1:** Effect of Eugenol supplementation on containing *Salmonella* colonization in chickens and spread.

Treatment Method	Effect	Reference
Eugenol (250 ppm) supplemented commercial feed	Prevented *S.* Enterica cross-contamination in eggs and prevented intestinal colonization by *S. enterica*	[95]
Eugenol treatment of chicken cecum in vitro model at 50 at 75 mM conc.	Reduced *S.* Enteritidis to <1.0 log^10^ cfu/mL (*p* ≤ 0.05) in chicken cecum	[92]
Feed supplementation with 0.75 and 1% Eugenol	≥3 log^10^ CFU/g reduction (*p* < 0.05) of *S.* Enteritidis in cecum	[94]
1% Eugenol supplementation in feed	1.5 log^10^ cfu/g reduction of *S*. *Enterica* in cecum and 2 log^10^ cfu/g in clocoa from >4 log^10^ cfu/g. Both results were statistically significant (*p* < 0.05)	[86]
In vitro application of subinhibitory concentrations of eugenol on chicken oviduct epithelial cells (COEC)	Highly significant reduction (*p* < 0.01) in *S.* Enteritidis colonization of COEC. The results have significance for the control of *S.* Enteritidis colonization of eggs	[96]
1/2 MIC eugenol pretreatment of chicken	Significantly decreased (*p* < 0.01) *S.* Typhimurium loads in various organs. It also improved chicken survival rate and weight gain at 1/2 eugenol MIC	[85]

## Data Availability

Not applicable.
